# Telehealth Access and Substitution in the VHA

**DOI:** 10.1007/s11606-023-08465-0

**Published:** 2024-02-23

**Authors:** Jessica Lum, Izabela Sadej, Steven D. Pizer, Christine Yee

**Affiliations:** 1 VA Boston Healthcare System, Boston, MA USA; 2 Department of Health Law, Policy and Management, Boston University of Public Health, Boston, MA USA

## Abstract

**Background:**

In response to COVID-19, the Veterans Health Administration (VHA) expanded telehealth availability, allowing veterans to receive care at home. We explore the extent of substitution of telehealth for in-person care among medical centers (facilities), providers, and patients. We explore the extent to which patient preferences drive telehealth utilization, and compare access to care (as measured by waiting times) for telehealth and in-person visits.

**Methods:**

We use VHA electronic health records to identify scheduled outpatient mental health (MH) appointments from January 2019 through February 2023 focusing on care delivered by social workers, psychologists, and psychiatrists. For each quarter, we compute the proportion of completed appointments that were delivered via phone or video by each facility, provider, and patient and show the changes in these proportions before, during, and after the onset of COVID-19. To explore patient preferences, we match providers of patients with high rates of telehealth utilization and examine the extent to which those providers deliver in-person care. To examine access to care, we compute waiting times for in-person, video, and phone new patient appointments. We investigate differences between urban and rural patients, and patients of different ages.

**Key Results:**

Telehealth for MH grew dramatically in the VHA after the onset of COVID-19. While some facilities provided more telehealth than others, all facilities (as of early 2023) provided some telehealth MH services. Approximately 86% of individual providers provided telehealth, with 27% scheduling MH appointments almost exclusively as telehealth appointments and 59% providing a mix. Patients exhibited more polarization, with 36% scheduling only in-person visits for almost all their MH visits and 56% of them scheduling exclusively telehealth, and only 8% of them utilizing a mix of modalities. Of those who exclusively received telehealth care, a majority of them utilized video (80%) over phone (20%). Take-up of MH among younger patients was higher relative to older patients. Urban patients used telehealth more than rural patients. Patient preferences rather than provider preferences drove utilization of patients who almost exclusively utilized telehealth. Between April 2021 and February 2023, the average difference in waiting time for in-person and video appointments was less than 1 day, with comparable appointment volumes, suggesting that the supply of and demand for in-person and video were not different enough to merit waiting longer. Telehealth was chosen over in-person more among urban and younger patients, as older and rural patients exhibited higher willingness to wait for in-person over video appointments. By contrast, appointment volumes and waiting times for phone appointments were lower across all groups, suggesting that phone may not be as substitutable for in-person visits in MH.

**Conclusions:**

We find that the VHA has made telehealth widely available, providing access to many veterans. While telehealth utilization has increased, face-to-face care persists for MH services, suggesting that one modality may not serve all purposes and preferences for care. Patient preferences drive the modality decision among those who exclusively use MH care via telehealth. For those who persist in mostly utilizing in-person care, there may be various factors influencing those preferences such as issues with limited internet connectivity, language barriers, and digital literacy, especially for older and rural patients who utilize in-person care more than those who are younger and more urban. Further investigation is required to investigate the optimal mix of modalities which may allow for potential increases in patient satisfaction, quality of care, and clinic efficiency.

**Supplementary Information:**

The online version contains supplementary material available at 10.1007/s11606-023-08465-0.

## INTRODUCTION

The onset of the COVID-19 pandemic presented a unique challenge for health care delivery systems, requiring an immediate shift away from in-person patient care in most settings. This served as the catalyst for telehealth services, which allowed providers to continue providing care in a safe manner^1^. The Veterans Health Administration (VHA), under the leadership of the U.S. Department of Veterans Affairs, took rapid action to promote the use of telehealth modalities throughout the pandemic. This was done through initiatives that expanded telehealth infrastructure, spread messaging on the availability of telehealth to patients, increased training for providers, and relaxed restrictions on the use of HIPAA-compliant video platforms.^1^

VHA has been a pioneer in the adoption of virtual health care services, with implementation beginning in 2003 to reduce barriers to access for veterans with the Care Coordination Home Telehealth (CCHT) program, which provided telehealth services to veterans with chronic conditions, laying the foundation for further telehealth expansion in the VHA over the following decades.^2^ In 2018, as part of the VHA Maintaining Internal Systems and Strengthening Integrated Outside Networks (MISSON) Act, the VHA established the “Anywhere to Anywhere” telehealth initiative to ensure that all VHA health care providers in outpatient mental health (MH) settings and in primary care service lines are able to provide telehealth services to veterans’ homes by 2021.^3^ Any veteran who qualifies to receive VHA health care benefits and lives within the USA is eligible to use VHA telehealth services, which includes VA Video Connect (referred to as video care) and phone care.^4^

Previous studies have found that in June 2020, 58% of VHA care was provided by telehealth, compared to 14% prior to the onset of the pandemic in March 2020.^1^ With increasing rates of care delivered via telehealth, it is important to examine the degree to which in-person visits have been substituted with telehealth. We define substitution as the practice of delivering/scheduling visits remotely through telehealth technologies (phone or video) instead of in-person for similar types of visits. This study examines access to video and phone care and the extent to which these modalities have been substituted for in-person care in VHA mental health settings. Understanding the extent to which different patients and their providers substitute one modality for another may inform future healthcare policy and strategy, allowing for optimized healthcare delivery and accessibility. Investigating substitution may guide how telehealth resources should be targeted and capacity planning.

As patients utilize telehealth as substitutes for in-person care, they may save time associated with traveling to see the provider, and providers may save physical space.^5, 6^ In addition, the risk of contagion may be reduced while still maintaining the patient-provider connection.^7^ However, as telehealth capabilities expand, disparities in access may be exacerbated as limited Internet bandwidth can prevent certain communities from being able to access and receive care via video.^8^ Despite the rapid overall increase in the use of telehealth, not all patients and providers have adopted it equally. Certain patient populations, such as older adults living in rural areas, may be less capable or willing to participate in video telehealth visits,^9^ while others report video care being equivalent to in-person care.^10^

From a clinic-level management perspective, video and phone care may potentially result in higher efficiency. Providers may be able to provide more visits per day if fewer telehealth appointments are cancelled last minute than in-person visits. Telehealth also allows for providers elsewhere (in particular, those who have smaller caseloads) to provide care in areas where providers are scarce. If telehealth ultimately allows treating more patients (with a given health concern) with the same number of provider hours, and substitution does not yield worse outcomes or patient satisfaction, it should be encouraged over in-person care (for those types of patients and health concerns).

The growth in and adoption of telehealth utilization for mental health care was particularly rapid in the VHA during the pandemic, which may be due to the existing telehealth infrastructure and earlier efforts to promote video mental health care.^11, 12^ Relative to other specialties, mental health may be more amenable to substitution with telehealth. Examples include initial mental health assessments and evaluations, routine check-ins and follow-ups, therapy and counseling, medication management, peer support groups, and psychoeducation, as well as crisis intervention for individuals who need immediate support. On the other hand, there are some medical examinations, diagnostics, and treatments that need to be performed in-person for which virtual modalities cannot be a substitute. While much of the previous literature has documented overall trends in care delivery across different modalities of care, we provide a new perspective by describing variations in patterns of substitution of visits within individual facilities, providers, and patients. By grouping patients by their visit patterns and examining the patterns of their associated providers, we can observe patient preferences for receiving care via one modality over another. If a patient who regularly uses VHA for MH services was observed as having all their visits via telehealth, and we observed that the patient’s providers mostly delivered care in-person, it would suggest that the patient preferred telehealth over in-person visits. In addition, we analyze the uptake of telehealth among the overall population of mental health patients at the VHA as well as utilization among urban vs. rural patients, among different age groups, as well as different provider types to assess potential disparities in telehealth adoption.

We also document the fluctuations in waiting times and volume for telehealth and in-person visits. Waiting times together with volume inform us not only about access to each modality of care but also about supply of and demand for one modality relative to another.^13^ If one modality has higher waiting times than that of others, this suggests a more limited supply relative to demand for that modality. Similar waiting times suggest the marginal patient is indifferent between modalities.

## DATA SOURCE AND STUDY POPULATION

We use the VHA’s Corporate Data Warehouse (CDW), a repository of electronic health records for veterans. Our sample period spans from January 1, 2019, to February 28, 2023. VHA’s Managerial Cost Accounting Stop Codes allow the VHA to identify different types of health care services. Stop codes also allow for identification of modality of care delivered (in-person, video, phone). We used stop codes to restrict our sample to mental health services and to categorize encounters and appointments by modality of care delivery^1^ (Supplementary Tables 1, 2). We restrict our sample to outpatient mental health encounters and appointments that were video, phone, or in-person, excluding secure messaging, chart consults, and asynchronous records. We also exclude outpatient visits that occurred while a patient was hospitalized. We exclude records from five facilities that converted their electronic health record systems to Cerner in order to exclude potential inconsistencies in data collection across facilities that did and did not transition.

For analyses of utilization patterns of individual facilities, providers, and patients, we focus on completed scheduled visits, excluding unscheduled visits or walk-ins, as scheduled visits may represent revealed preferences for a given modality more accurately than unscheduled visits. Our focus on scheduled visits is also driven by the fact that these visits may be more important in informing efficient allocation of staffing to maintain predictability in day-to-day operations, which may then improve the overall efficiency of a given clinic. In addition, we restrict this sample to visits from three provider types: social workers, psychologists, and psychiatrists. These three provider types accounted for 73% of all completed scheduled MH visits during this period. Focusing on these main provider types allows us to build a more homogenous sample to draw inferences on substitution. This sample consisted of 32,122,767 visits across 2,521,647 patients, 24,820 providers, and 135 unique facilities. To analyze utilization, availability, and substitution of each modality across facilities, we compute the proportion of total quarterly scheduled visits at each facility that were delivered via in-person, phone, or video. We compute a similar proportion of quarterly visits across modalities for individual providers and patients, and investigate utilization patterns for specific provider types, for urban vs. rural patients, and for patients aged 18–39, 40–64, and 65 and older We refer to instances where in-person visits account for 90–100% of all visits as “almost exclusively in-person,” 0–10% as “almost exclusively via telehealth.” Facilities, providers, and patients with between 10 and 90% of all visits occurring in-person are referred to as using a mix of modalities.

When investigating patient preferences, we first focus on 352,992 “consistent users” of MH in the VHA. We define these patients as those who had at least the average number of total MH visits observed in that calendar year (6 in 2019, 5 in 2020, 2021, and 2022). While these patients represented about 14% of patients in our sample, they accounted for 17,855,048, or 56%, of all the visits captured. Focusing on the patterns of utilization of these patients allows us to differentiate between substitution of one modality for another versus visits from patients who sought MH care only periodically and may not have strong preferences for one modality over another compared to consistent users. We further selected the sample to consistent users of MH who exclusively received care via telehealth, we matched these patients to their associated providers, and observe the patterns of care delivery of those specific providers. If a consistent user of MH who only schedules telehealth visits is observed to have providers with patterns of delivering most of their care in-person, it suggests that the patient prefers that modality, despite the history of care delivery by their providers.

Waiting times can provide insight into patient preferences when substituting one modality over another. We examine the waiting times for new patient appointments scheduled with an appointment date between January 1, 2019, through February 28, 2023. Appointments that may have been cancelled or were no-show were also included. Omitting those appointments could erroneously bias the waiting times downward since cancelled appointments may be associated with higher waiting times. New patients to mental health are defined as those who had no scheduled VHA mental health appointment in the past 3 years. Only the initial appointment for these new patients were counted. The waiting time is defined as the difference between the appointment date and the date when the appointment was created. We then calculate the monthly average waiting time of these new patient appointments as well as the monthly total new patient appointments scheduled for each modality. We focus on the waiting times for new patients as opposed to established patients, or all patients, because they have been shown to be highly correlated with patient satisfaction with access to care (at least in primary care and specialty care settings). Waiting times measured with other samples (e.g., established patients) or using other date variables do not seem to be consistent with satisfaction in access to care.^14^.

## RESULTS

**Figure 1:**
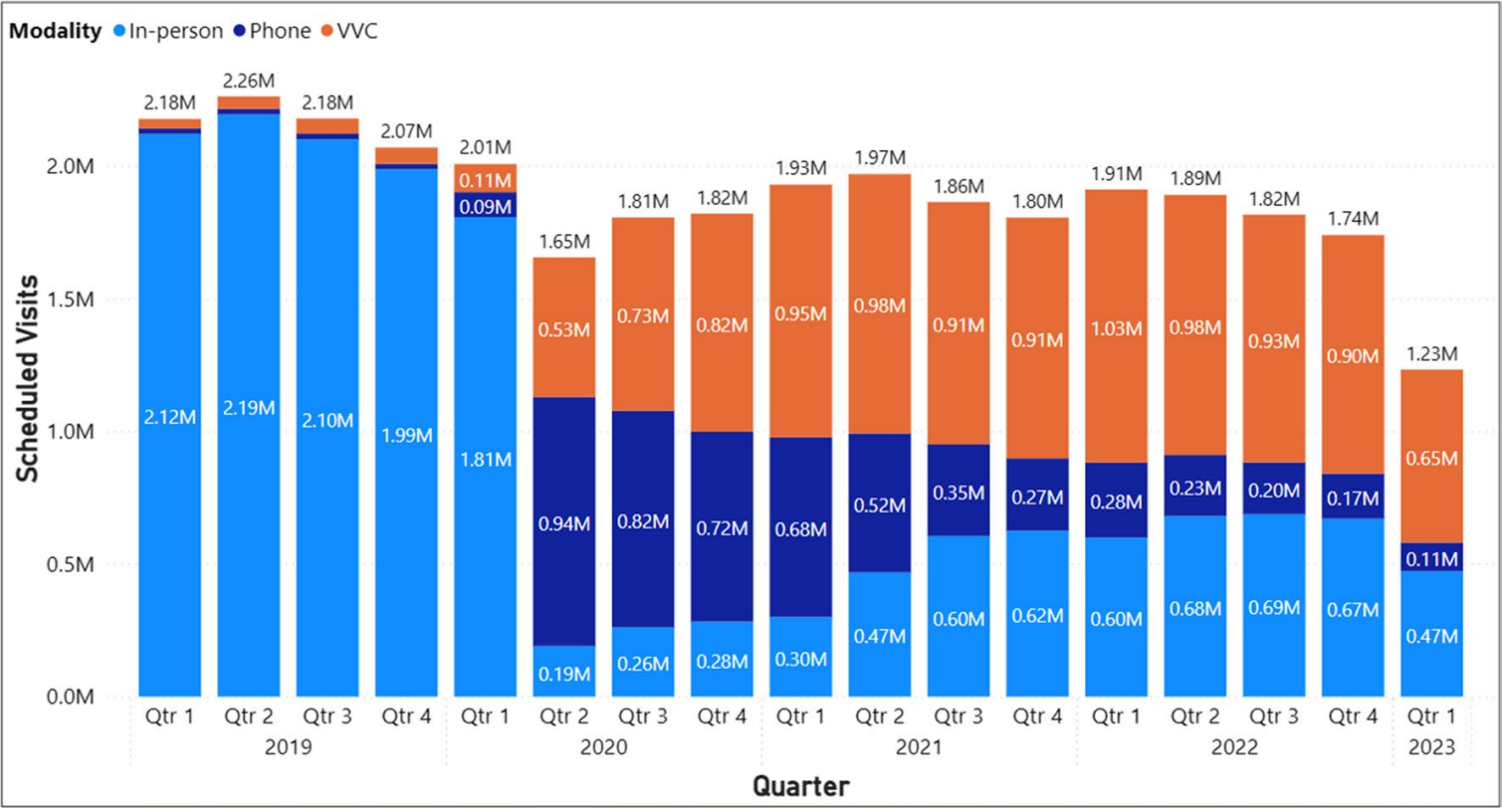
Total completed scheduled outpatient MH visit volume by modality (psychologists, social workers, psychiatrists).

Total scheduled MH visits decreased from 2.26 million in the second quarter of 2019 to about 1.97 million in the same quarter of 2021 (− 13%) and further declining to about 1.89 million in 2022 (− 16%). At the same time, the composition of these visits substantially changed relative to the pre-COVID time period (Fig. 1). Telehealth capabilities existed prior to the pandemic, representing just 3% of all MH visits (1% video and 2% phone). As phone visits do not require the same infrastructure or training requirements as video visits, they represented the majority of all visits at the start of the pandemic (about 57% of visits as of the second quarter of 2020), but decreased substantially, representing just 12% of visits by the same quarter of 2022. While the use of phone visits decreased, the VHA expanded capabilities for remote video care, and by the second quarter of 2022, video care represented over half (52%) of scheduled MH visits. In-person visits started to bounce back since the start of COVID, however representing only about 36% of scheduled visits in the second quarter of 2022, compared to 97% in 2019.

**Figure 2:**
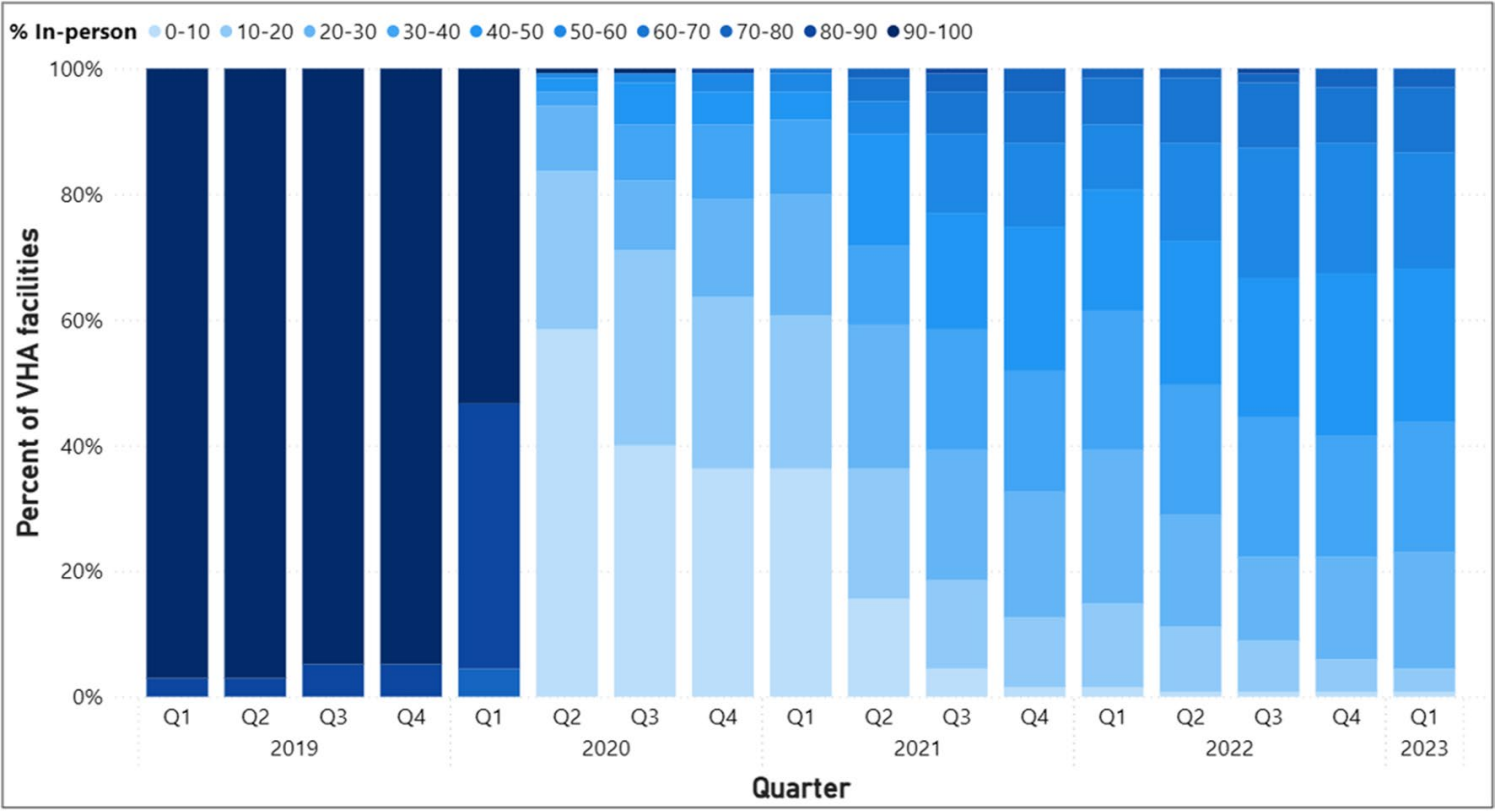
Percentage of facilities with varying levels of scheduled in-person visits as a percent of all MH visits.

The substantial changes in overall trends of visits across modalities may be explained by telehealth adoption that occurred within individual facilities. In 2019, more than 95% of all facilities had between 90 and 100% of scheduled mental health visits occurring in-person (referred to as “almost exclusively in-person”) (Fig. 2). In the second quarter of 2020, we observed the largest share (59%, or 79 facilities) of facilities with between 0 and 10% of scheduled mental health visits occurring almost exclusively in-person. We refer to these facilities as those who had visits “almost exclusively via telehealth.” There were 34 facilities (25%) with between 10 and 20% visits occurring in-person, 14 (10%) facilities with between 20 and 30% in-person, and 7 facilities (4%) with between 30 and 50% in-person. Only 1 facility had visits almost exclusively in-person. One year later, all facilities across the VHA delivered mental health services using a mix of in-person and virtual modalities and by Q2 of 2022, facilities were more evenly distributed across the levels of in-person care delivered with only one facility providing care exclusively via telehealth, and the rest providing a mix of modalities, with the largest share of facilities (31, or 23%) providing between 40-50% of care in-person.

**Figure 3:**
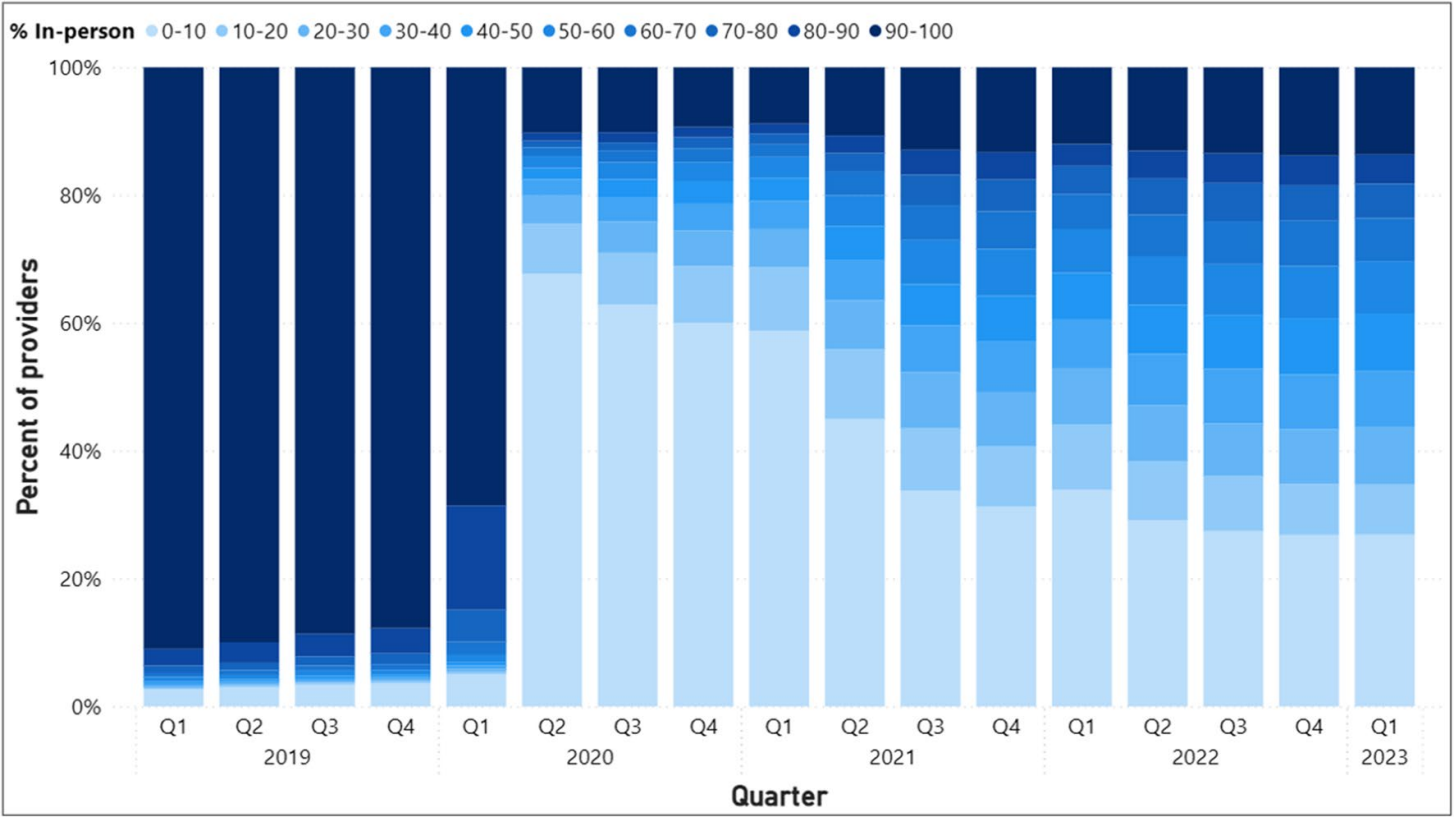
Percentage of providers with varying levels of scheduled in-person visits as a percent of all MH visits.

The growing share of facilities with intermediate (between 20 and 90%) levels of in-person utilization can be explained by patterns seen among individual providers with those same levels of in-person utilization (Fig. 3), which accounted for 22% of all providers in Q2 of 2020 but increased substantially to about 59% of all providers by the end of 2022. This increasing share of providers offering a mix of modalities overtime coincides with a decrease in the share of providers delivering care exclusively via telehealth. About 68% of providers in Q2 of 2020 delivered care almost exclusively via telehealth with continued decreases over time such that these providers represented 27% of all providers by the end of 2022. In contrast, the share of providers who delivered care exclusively in-person seems to be relatively stable. In Q2 of 2020, only about 10% of providers exclusively delivered care in-person, delivering care mostly via telehealth, and only slightly increased to 11% in Q2 of 2021, and 14% of providers by the end of 2022. As the share of providers offering a mix of modalities grew while the share of providers who delivered care almost exclusively via telehealth decreased, the relatively stable levels of providers who delivered care almost exclusively in person suggests that some providers continue to be reluctant in offering care using telehealth.

Different provider types may have mutually exclusive roles and responsibilities when caring for mental health patients. Social workers focus on holistic support and can provide coordination of care while psychologists may provide psychotherapy and counseling, and psychiatrists may prescribe medications and assist with medication management. Because of this, analyzing provider patterns specific to each of these provider types may provide additional insight into substitution of one modality for another for the similar types of services. We find qualitatively similar patterns of the distribution of providers across degrees of care provided in-person when looking at patterns for each provider type (Appendix Figs. 1, 2, 3) when compared with the patterns of in-person care delivery for all providers seen in Fig. 3.

**Figure 4:**
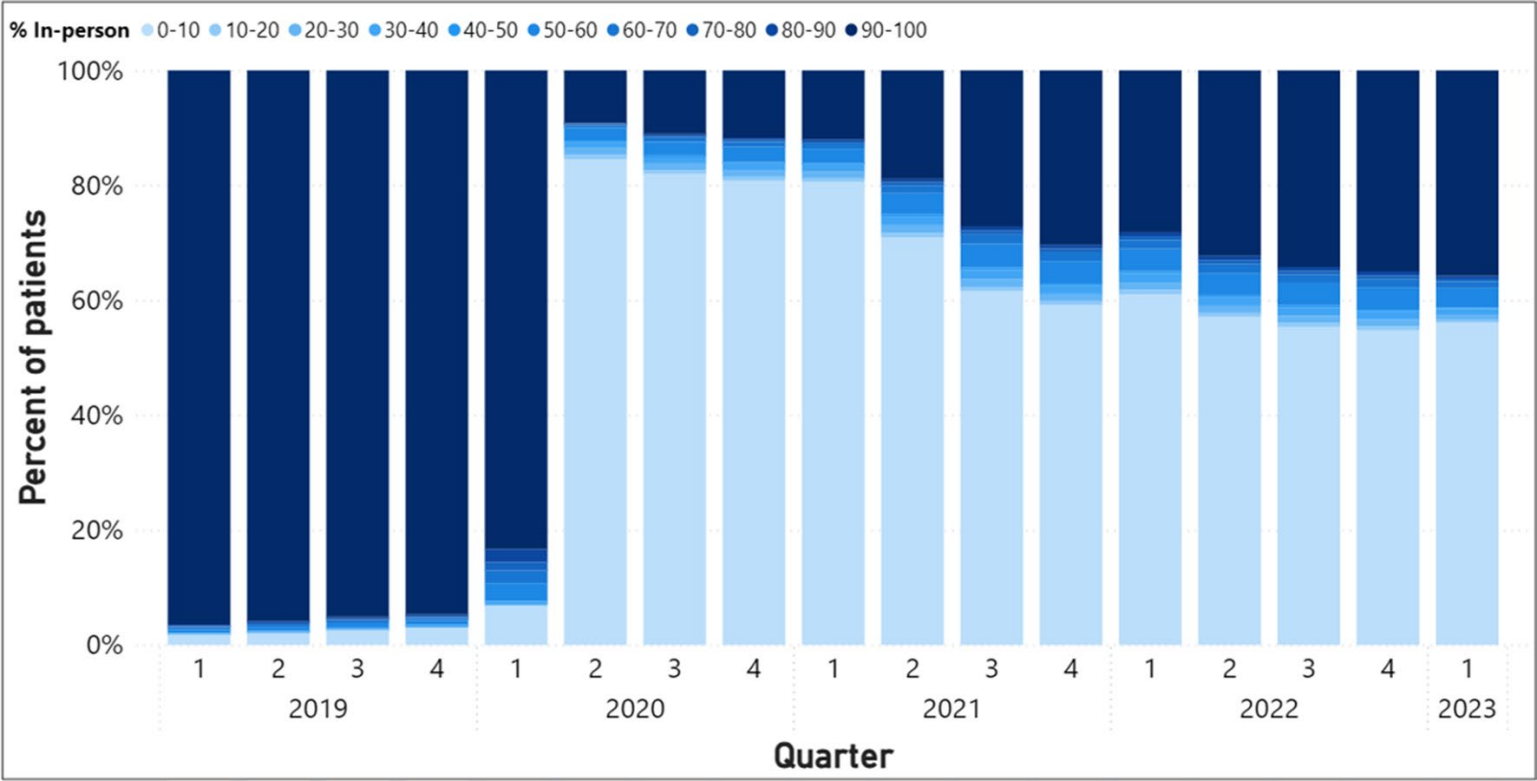
Percentage of patients with varying levels of scheduled in-person visits as a percent of all MH visits.

Relative to providers and facilities, the distribution among patients is more dichotomous (Fig. 4). The data suggest that patients exhibited a strong preference for either in-person or telehealth visits. Only 6% of patients scheduled a mix of in-person and telehealth visits in the second quarter of 2020 rising modestly to about 8% of patients by the early of 2023 (as shown by the moderate blue colored bars). The remaining patients scheduled either exclusively in-person visits or video/phone visits.

An increasing share of patients received care almost exclusively in-person in recent months relative to the start of the pandemic (35% of patients as of Q4 2022 vs. 9% of patients in Q2 of 2020). Despite the decreasing share of patients receiving care almost exclusively via telehealth, they continued to represent a majority (55%) of MH patients by the end of 2022.

We find similar dichotomies within certain groups of patients, although the degree to which in-person visits are chosen varies across groups. Patients aged 18–64 utilized more mental health services over time relative to those aged 65 and older; however, we find that a larger percentage (31%) of older patients utilize in-person mental health care relative to younger counterparts aged 18–39 (19%) and 40–64 (25%) as of the last quarter of 2022 (Appendix Fig. 4). Patients who lived in rural areas seemed to choose in-person a little more often as well (30% rural vs. 27% urban) (Appendix Fig. 5). Despite this, patients who received care exclusively via telehealth represented the largest share of urban (59%) and rural patients (58%), as well as patients in each age group (66%, 61%, and 57% of patients aged 18–39, 40–64, and 65 + , respectively).

We examine utilization patterns of consistent users of MH (Appendix, Fig. 6) which were similar to the trends observed for all patients (Fig. 4). As of the fourth quarter of 2022, about 29% of all consistent users exclusively received care as in-person visits, and 54% of all consistent users exclusively received care via telehealth. If patients who exclusively had their visits via telehealth were mostly seen by providers who also delivered most of their MH care using telehealth, this may reflect similar preferences by both the provider and patient for telehealth visits. However, for patients who exclusively had their visits via telehealth, about 9% of their providers delivered care almost always in person, and 63% of all their providers delivered care using a mix of modalities (Appendix, Fig. 7). This suggests that telehealth utilization for these patients were driven by patient preference rather than provider preference.

As a sensitivity analysis, we capture all completed scheduled visits for patients who are relatively new and did not see the VHA for MH care in the past 3 years (new patients), and for patients who were already established (Appendix Figs. 8, 9). In Q4 of 2022, more than half of new and established patients exclusively received care via telehealth (57% and 52%, respectively). For patients who almost exclusively used telehealth for MH, we observe similar trends for providers of the new and established patients as we do for the providers of consistent users of MH, in which 63% of their providers delivered care using a mix of modalities, while 10% of their providers delivered all their care in-person (as of 2022, Q4), presumably because there is substantial overlap in the providers who serve these different groups of patients (Appendix Fig. 10, 11).

**Figure 5:**
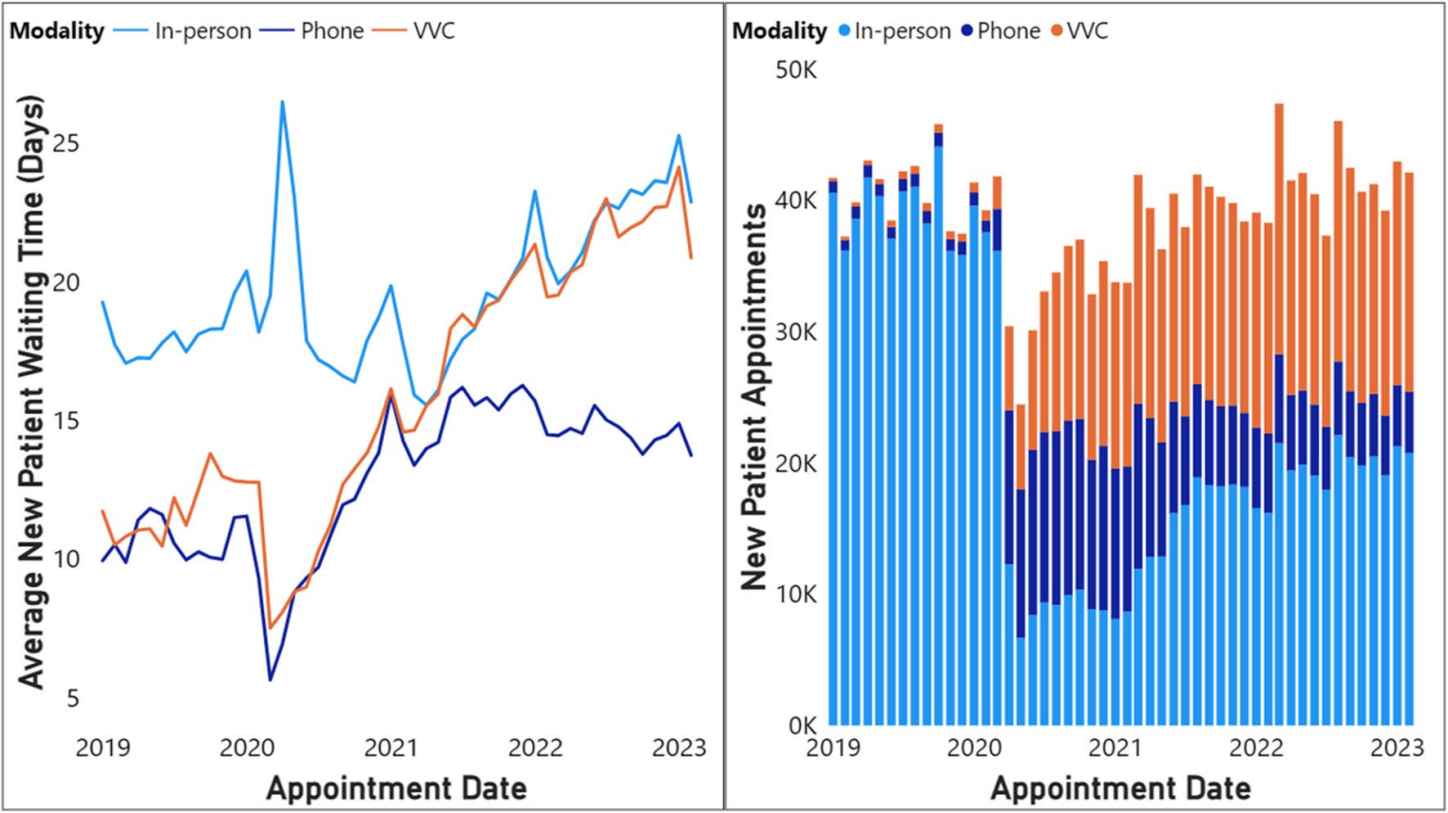
Average new patient waiting time (left) and monthly new patient appointment volume (right).

As the VHA rapidly responded at the start of the pandemic with a reduction in in-person visits, this triggered a spike in in-person waiting times (Fig. 5). Waiting times for phone and video visits were approximately 10 days prior to the pandemic and increased to between 13 and 15 days, respectively, by March 2021. By July 2021, phone care waiting times decreased from about 16 days to 14 days in Februarv 2023 while waiting times for video care increased and started to mirror the waiting times for in-person appointments. By February 2023, waiting times for video and in-person appointments both grew to similar levels, with a difference of less than 1 day on average since April, 2021. As these waiting times are calculated using all scheduled appointments, regardless of whether they were completed, their similar waiting times suggest that the marginal patient’s willingness to wait for an in-person visit was equal to that for a video visit. The preference for in-person relative to video was not different enough to merit waiting longer (given the supply of each modality). Examining waiting times of new patient appointments across patients in different age groups, and across urban and rural patients reveals that the indifference between waiting for in-person and video care applies mostly to younger and urban patients. Between 2021 and 2023, rural new patients consistently scheduled relatively more in-person appointments and had larger differences in waiting times between in-person and video visits than urban patients (Appendix, Fig. 12, 13). Rural patients consistently prefer to wait for in-person appointments over video appointments, with a wait of 24 days vs. 19 days as of February 2023, respectively. Patients aged 65 and up scheduled more in-person than video appointments relative to their younger counterparts (Appendix Fig. 14). While patients under the age of 65 exhibited similar waiting times for video and in-person appointments, waiting times for older patients diverged, preferring to wait longer for in-person care than for video appointments (27 vs. 22 days in February 2023) (Appendix Fig. 15).

By contrast, appointment volumes and waiting times for phone appointments relative to the other two modalities suggests that phone visits may not be as preferred. Despite lower waiting times, new patients are not requesting phone visits over in-person and video.

## DISCUSSION

This study examines shifts in the modality of mental health services delivered in the VHA from January 2019 to February 2023. Several factors may be associated with variation in in-person care delivered across facilities such as the proportion of rural patients, degree of specialty services provided, and staffing levels. Despite this, by early 2021, all facilities transitioned to offer mental health telehealth visits. This is in stark contrast to findings by Cantor et al. (2022) that showed only 68% of non-VHA mental health treatment facilities offered telehealth nearly 1 year into the pandemic.^15^ The growing share of facilities offering intermediate ranges of in-person care reflects the growing share of providers offering a mix of modalities over time. The share of providers who deliver care almost exclusively in-person seems stable over time representing around 12–14% of all providers from 2022 to the start of 2023, suggesting that some providers may not wish to or be able to offer telehealth to their patients. In contrast, 27% of providers delivered care almost exclusively as telehealth. We examined utilization patterns separately for social workers, psychologists, and psychiatrists. The distributions of these providers across levels of in-person utilization are similar to those seen for all providers combined which suggests that in-person visits were substituted with telehealth visits for similar types of services.

On the demand side, most patients almost exclusively scheduled telehealth visits over in-person visits (56% vs. 36% of all providers in early 2023). Patients exhibited discrete preferences for receiving all their mental health services either in person or via telehealth with only 8% of patients scheduling a mix of modalities by early 2023. Some older patients may not wish to or be able to utilize telehealth as much as younger patients, with 31% of patients aged 65 and up receiving care exclusively in person compared to 25% of patients aged 40–64 and 19% of patients aged 18–39. The differences between rural and non-rural patients were not drastically different, although a slightly higher proportion of rural patients received in-person care over telehealth. Over half of patients in each of these subsets received MH care exclusively via telehealth. This suggests that telehealth is accessible for a large share of patients. But while decisions to choose one modality over another may reveal one’s preferences, the decisions are made given individuals’ constraints and environment, such as the lack of technology or bandwidth, differences in digital literacy, language barriers, and medical complexities.

Of patients who consistently used VHA for MH care each year, about 54% exclusively used telehealth by the end of 2022. These high utilizers of telehealth were mostly seen by providers who had a history of delivering care using a mix of modalities, representing about 63% of their providers, as well as providers who exclusively delivered care in-person, about 9% of their providers. We observed similar results for patients who were new to VHA for MH services as well as for all other patients with an established relationship with VHA for MH care. For these patients, substitution of in-person visits with telehealth visits was driven by patient preference rather than provider preference.

Overall, the convergence of waiting times and volume for video and in-person visits suggests that the patients at the margin are indifferent between in-person and video visits. However, starting in April 2021, waiting times for those modalities increased from 16 to about 23 days during this time, suggesting that the overall demand grew faster than the supply of VHA MH care. While urban and younger patients exhibited similar willingness to wait for in-person and video appointments, older and rural patients were more willing to wait for in-person over video appointments.

In terms of the type of telehealth, we found that phone was the dominant modality for scheduled visits at the start of the pandemic in 2020; however, a year later, it was the least common modality. Despite lower waiting times, phone visits do not seem to be scheduled as often and so may not be as preferred as video for telehealth. This suggests that phone visits may be less substitutable for in-person visits (that are scheduled ahead of time). As phone appointments are less resource intensive than video appointments, future research should seek to characterize instances in which phone visits may be an appropriate substitute for in-person visits, and to understand factors that contribute to scheduling video visits over phone visits.

While demand for video care has increased, demand for face-to-face care persists. This has several policy implications. Regulators and facility management leaders should not broadly assume that one modality is superior to another. A patient-centered approach to care, offering choice of modality, may be more appropriate. Our findings suggest that older and to some extent rural patients seem to choose in-person over telehealth. While patients may choose care to be delivered solely via one modality, there may be a mix of modalities that most efficiently treat a health condition, in terms of the number of visits required for treatment and maintaining quality and patient satisfaction. More research is needed to determine the optimal allocation of resources and which areas and patients could benefit most from an increase in access to telehealth or in-person visits.

From a clinic management perspective, the impact on the cost of providing virtual care remains unclear. While short-term costs to develop proper telehealth infrastructure may be high, long-term gains include saving physical space, reducing contagion, potentially increasing provider welfare, and potentially improving downstream costs. Future research is also needed to identify specific services and treatments (e.g., therapy, medication management, care coordination) that may allow for or benefit most from remote care delivery without sacrificing quality of care or patient satisfaction.

## LIMITATIONS

This article has several limitations. This study examines mental health utilization and access to telehealth services for veterans who seek health care in the VHA. The results may not be generalizable to a non-veteran population. The VHA is a national system that is very integrated relative to other health care systems. In addition, veterans are more likely to be male, White, with higher rates of mental illness than the national population. Furthermore, demand for VHA services (and observed utilization and waiting times) may be affected by whether telehealth is provided by non-VHA mental health providers. Exploring dynamics of the supply of and demand for VHA and non-VHA services that affect utilization and waiting times are beyond the scope of this study.

Another limitation is that we do not study patient outcomes or provider efficiency; thus, we cannot make normative statements about whether telehealth *should* be used more than in-person visits. Although we observe a large percentage of patients with an increased share of telehealth utilization, we do not examine whether patient health outcomes or satisfaction was altered due to changes in modality. Similarly, we did not evaluate preferences among providers for telehealth or whether it changes productivity.

Finally, we used waiting times for new patient appointments as a measure of access to care. While a measure specific to mental health has not been validated with patient satisfaction with access, an aggregate wait-time measure has been validated across all major medical subspecialties^14^. This measure also focuses on scheduled appointments, which may not be representative of patients seeking urgent mental health care. We provide some descriptive information about the differences in scheduled and walk-in/last minute visits by modality and find that in-person and phone are currently the modalities of choice for urgent issues (Appendix, Fig. 16). There may be a relationship between waiting times and walk-in volume; it is possible that with increasing waiting times for scheduled in-person and video appointments, patients choose to walk-in rather than wait for their scheduled appointment. More research is needed to explore this.

## CONCLUSION

This paper describes the rapid growth and persistent use of MH telehealth in the VHA. All VHA facilities previously delivered MH care almost exclusively in-person. However, in response to the COVID-19 pandemic, they rapidly responded by delivering care using a mix of modalities. Providers exhibited flexibility when scheduling appointments to cater to patients with discrete preferences for receiving MH care either in-person, or via telehealth. Non-urgent new patients scheduled more video visits over phone visits, despite phone visits having shorter waiting times. With similar waiting times for in-person and video appointments, patients’ choice of modality may be driven by individual preferences rather than differences in waiting times across modalities.

### Supplementary Information

Below is the link to the electronic supplementary material.

Supplementary file1 (DOCX 1.10 MB)

## Data Availability

Study data from the VA Corporate Data Warehouse are not approved for public release.

## Declarations:

The work was determined not to be research by the VA Boston Healthcare System’s Research and Development Committee and thus oversight was not required.
