# Supplementary material for: Telehealth Access and Substitution in the VHA
Source: J Gen Intern Med. 2024 Feb 23;39(Suppl 1):44–52. doi: 10.1007/s11606-023-08465-0 (PMC10937886; doi:10.1007/s11606-023-08465-0)
Supplement: Supplementary file 1 — Supplementary file1 (DOCX 1.10 MB) [file 11606_2023_8465_MOESM1_ESM.docx]

**Appendix:**


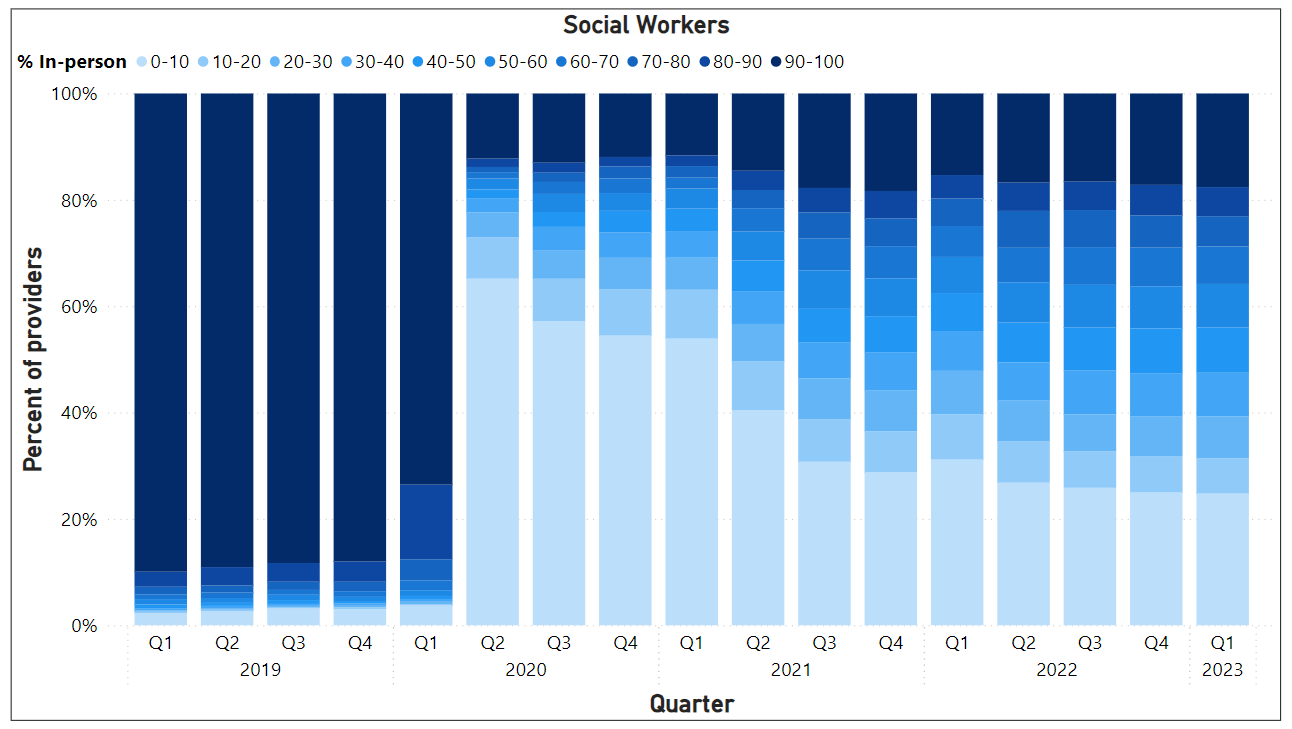


**Figure 1. Percentage of social workers with varying levels of scheduled in-person visits as a percent of all MH visits.**


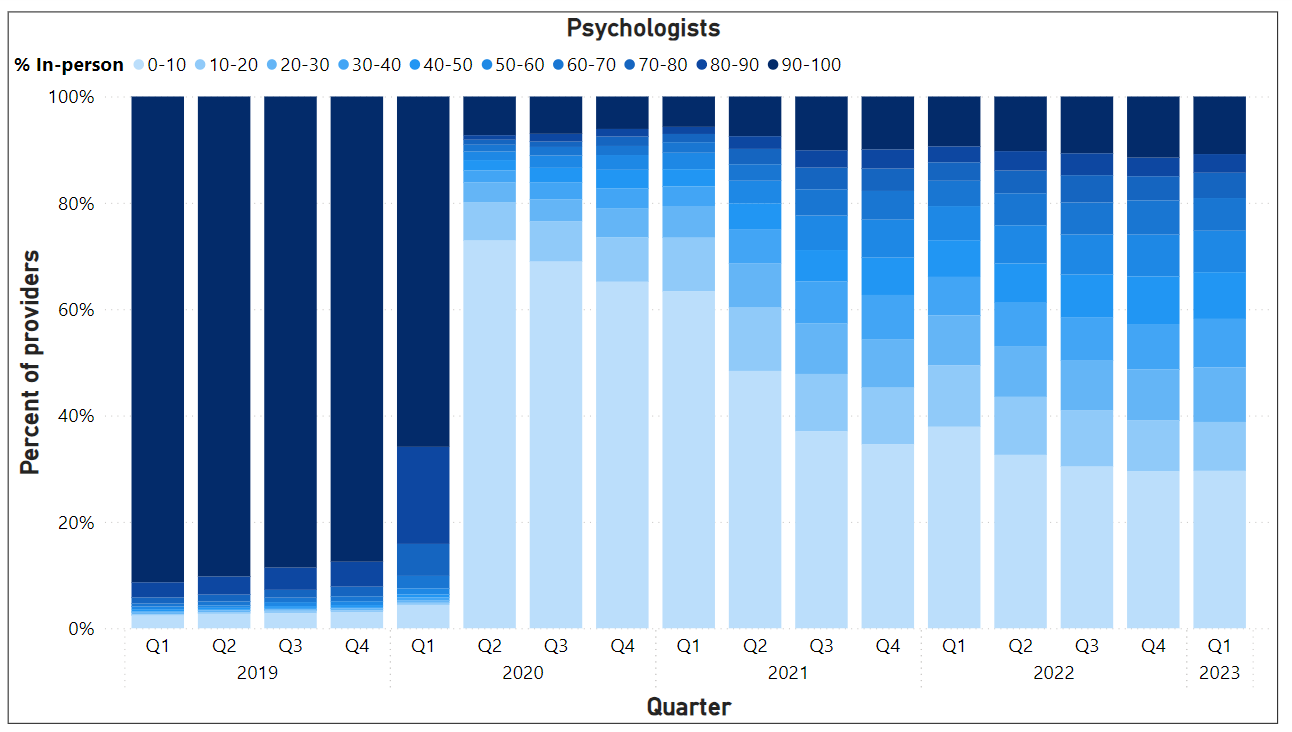


**Figure 2. Percentage of psychologists with varying levels of scheduled in-person visits as a percent of all MH visits.**


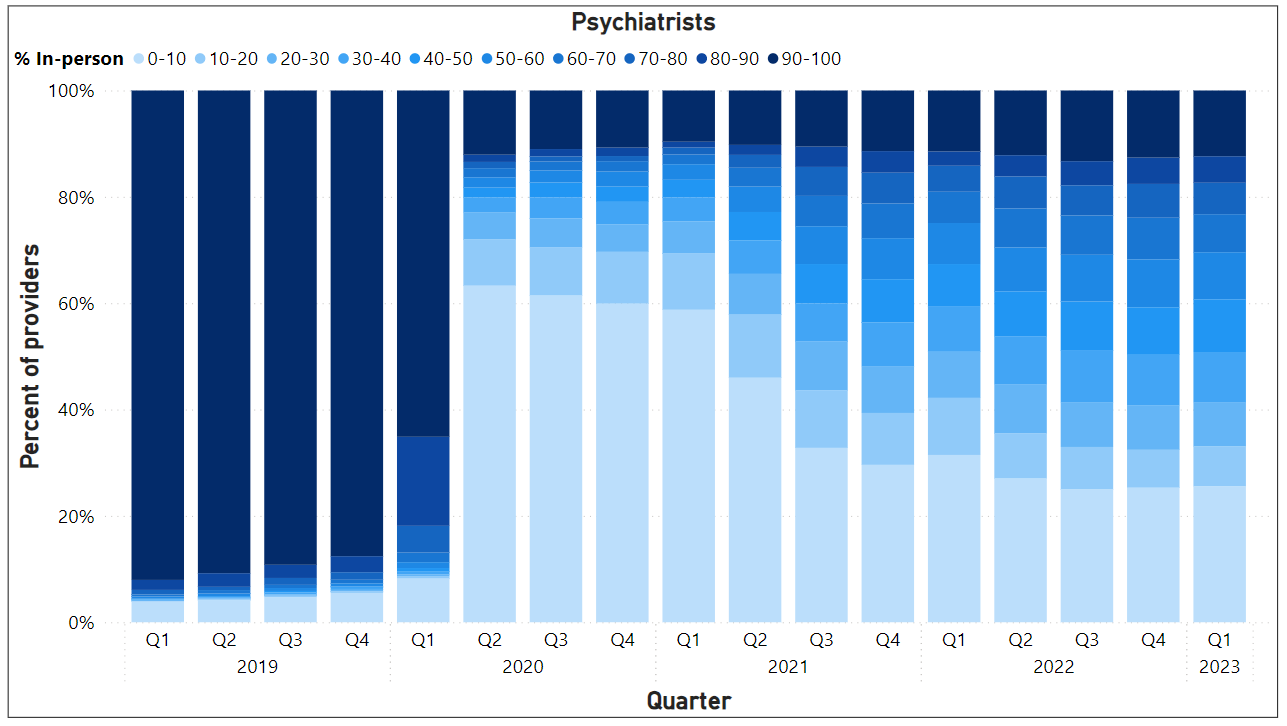


**Figure 3. Percentage of psychiatrists with varying levels of scheduled in-person visits as a percent of all MH visits.**


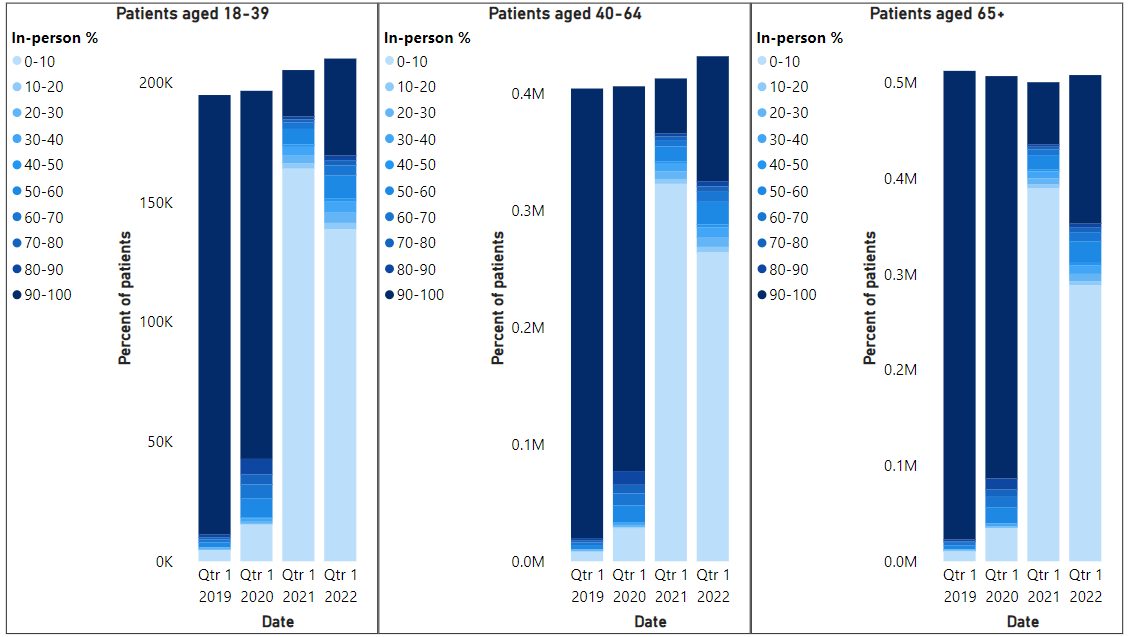


**Figure 4.** **Distribution of the percentage of patients across age group.**


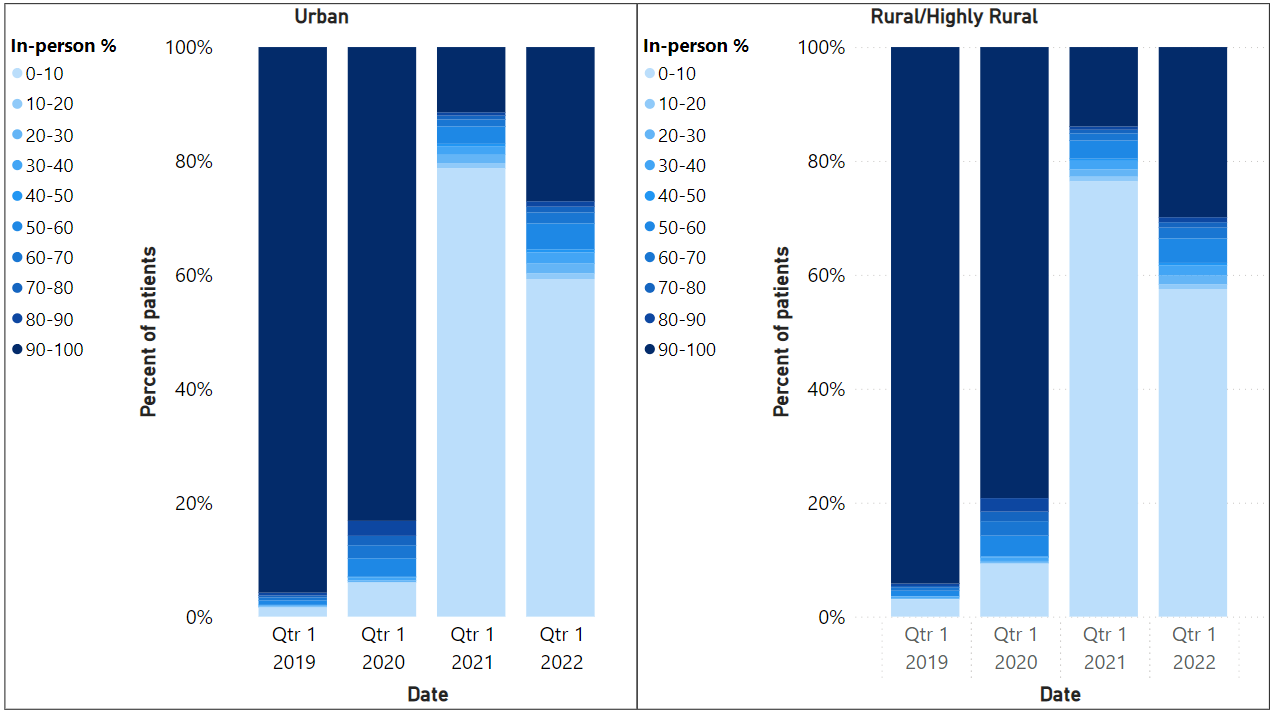


**Figure 5**. **Distribution of the percentage of patients across age group urban/rural status.**


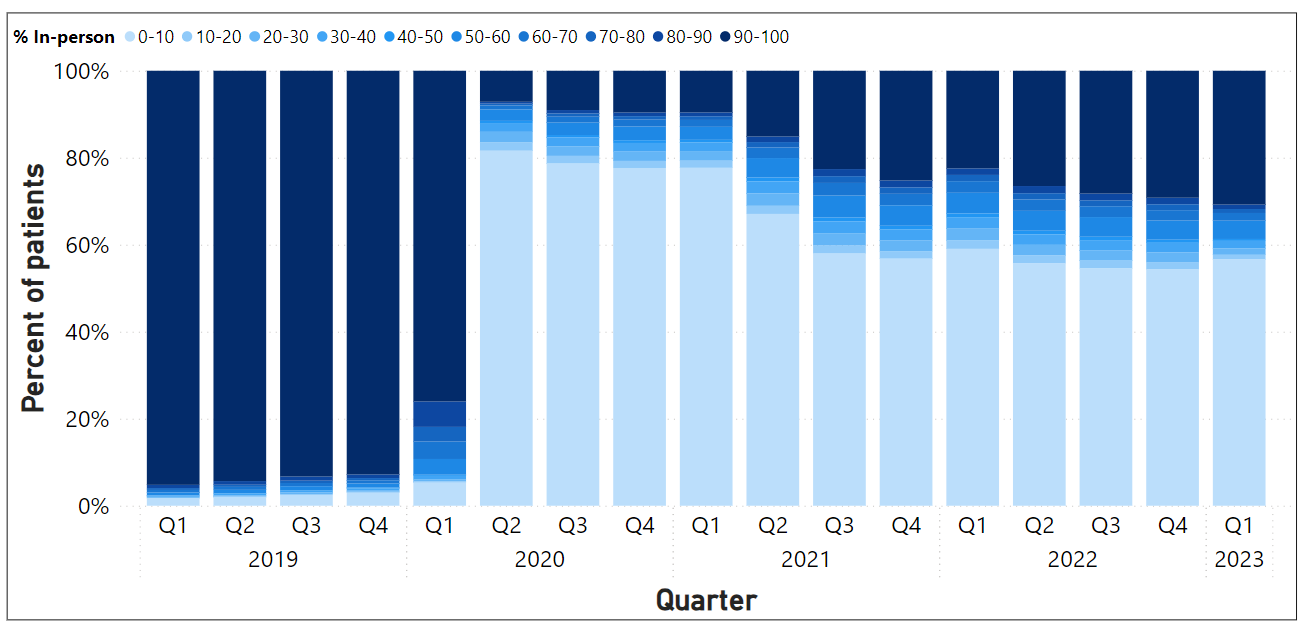


**Figure 6. Distribution of the percentage of patients across varying levels of in-person visits for consistent users of MH.**


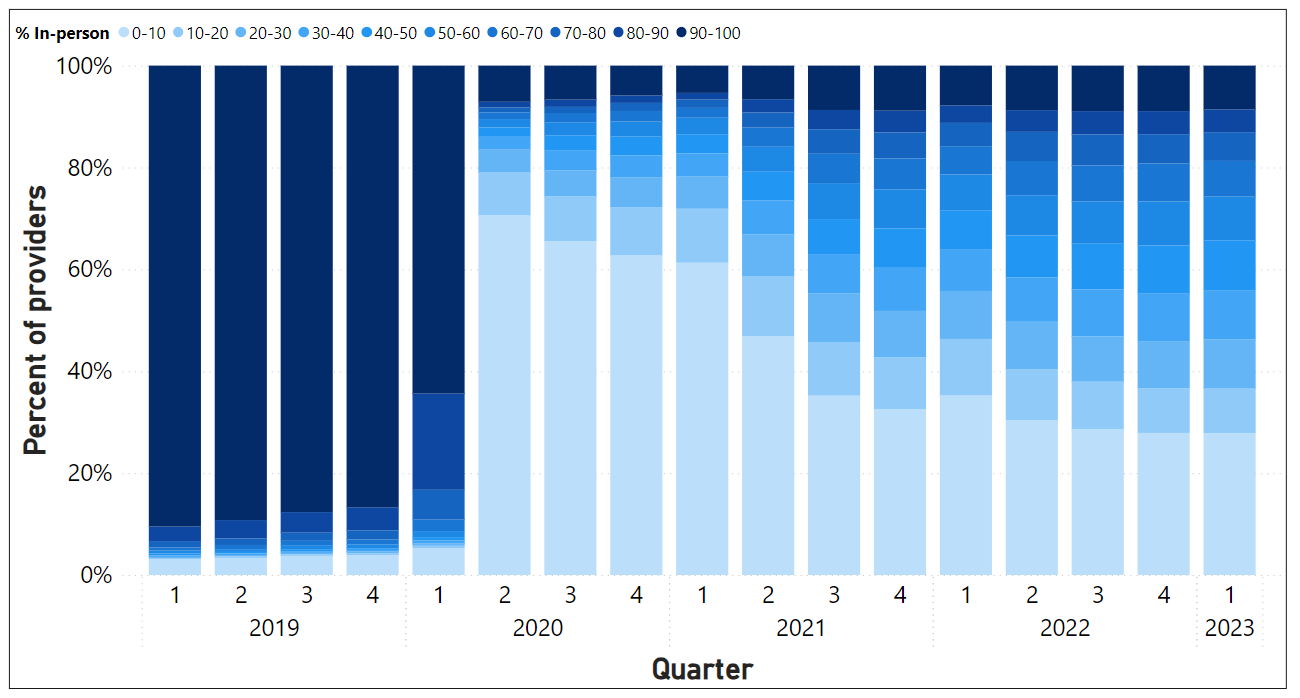


**Figure 7. Percentage of providers with varying levels of scheduled in-person visits as a percent of all MH visits (specific to consistent users of MH with 90-100% telehealth utilization).**


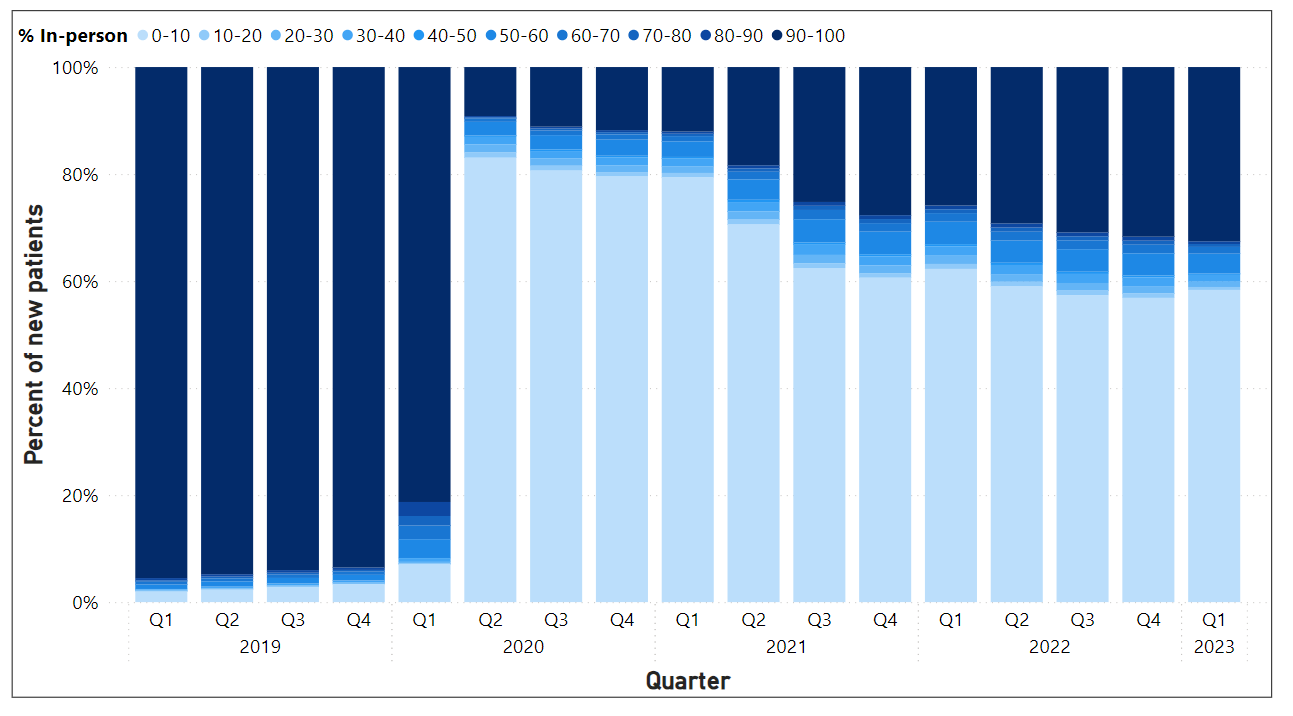


**Figure 8. Distribution of the percentage of new patients across varying levels of in-person visits.**


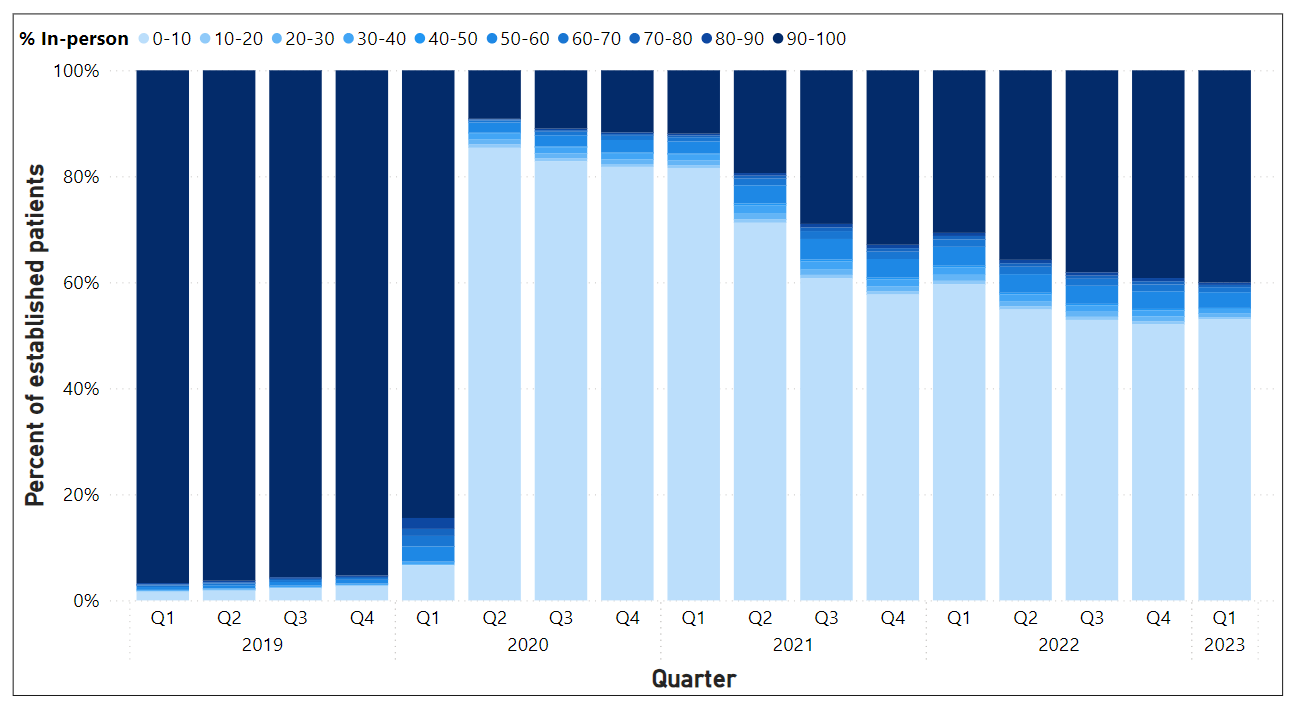


**Figure 9. Distribution of the percentage of established patients across varying levels of in-person visits.**


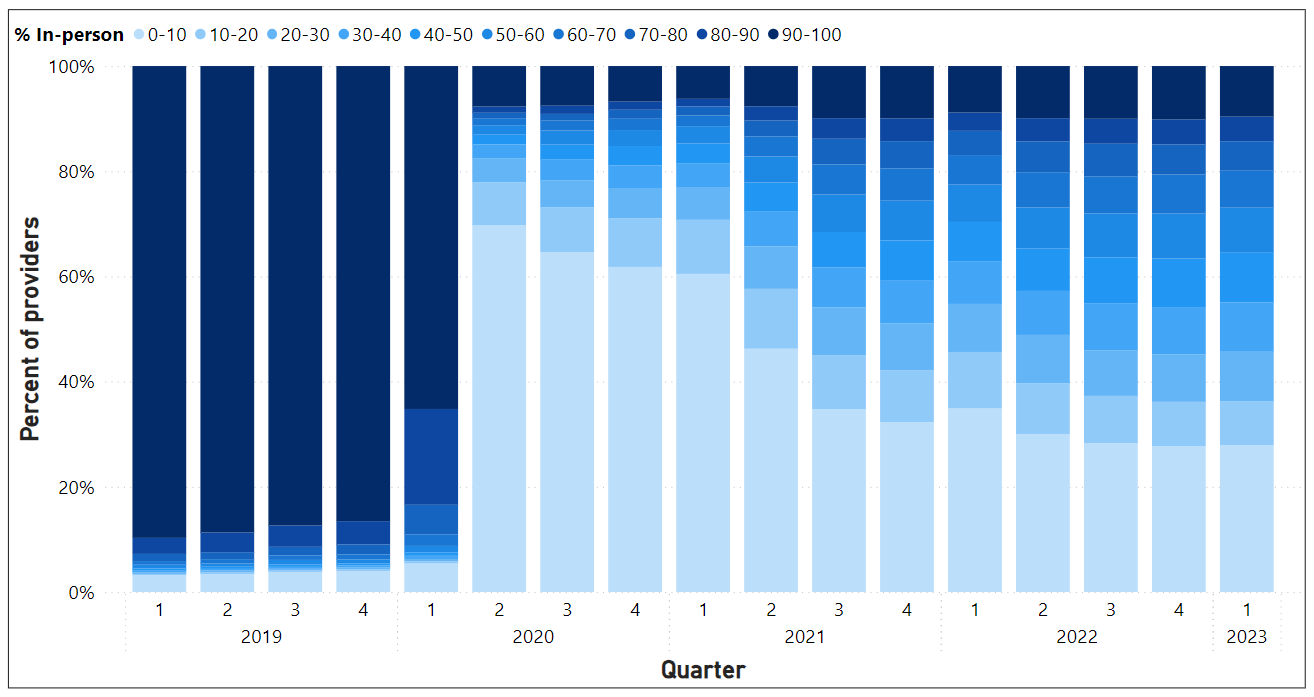


**Figure 10. Percentage of providers with varying levels of scheduled in-person visits as a percent of all MH visits (specific to new MH patients with 90-100% telehealth utilization).**


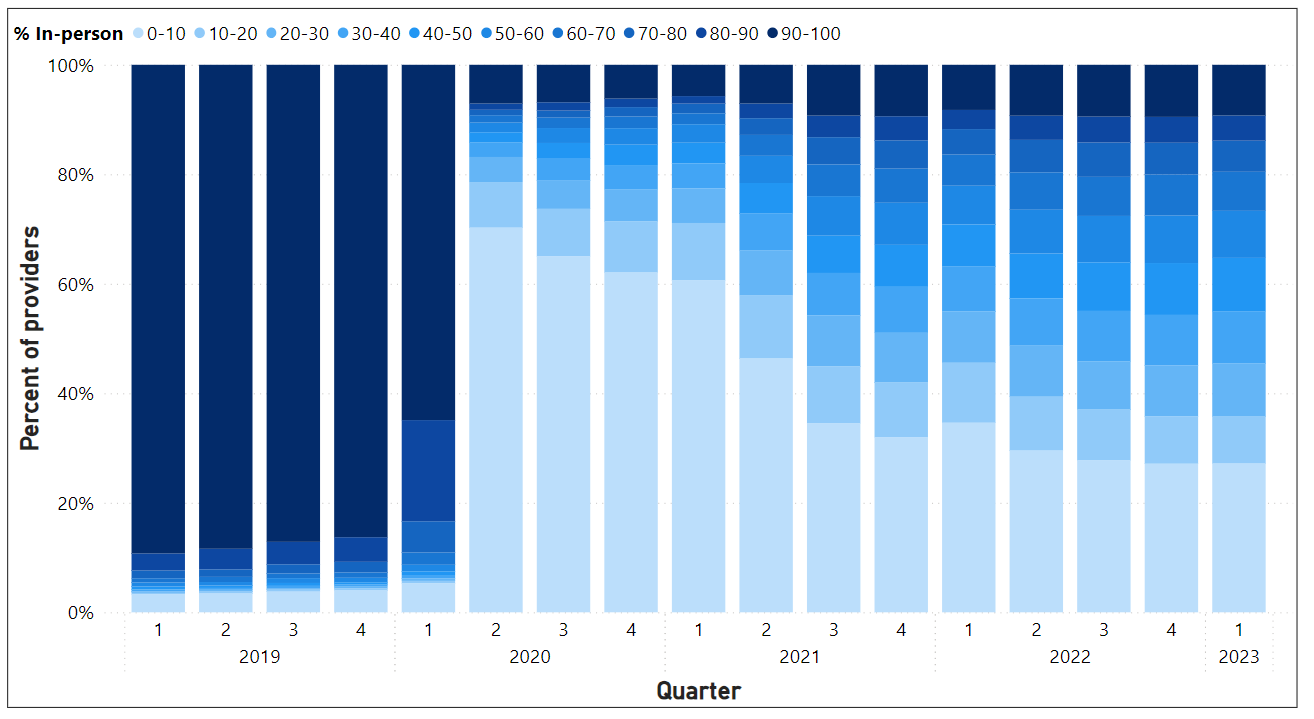


**Figure 11. Percentage of providers with varying levels of scheduled in-person visits as a percent of all MH visits (specific to established MH patients with 90-100% telehealth utilization).**


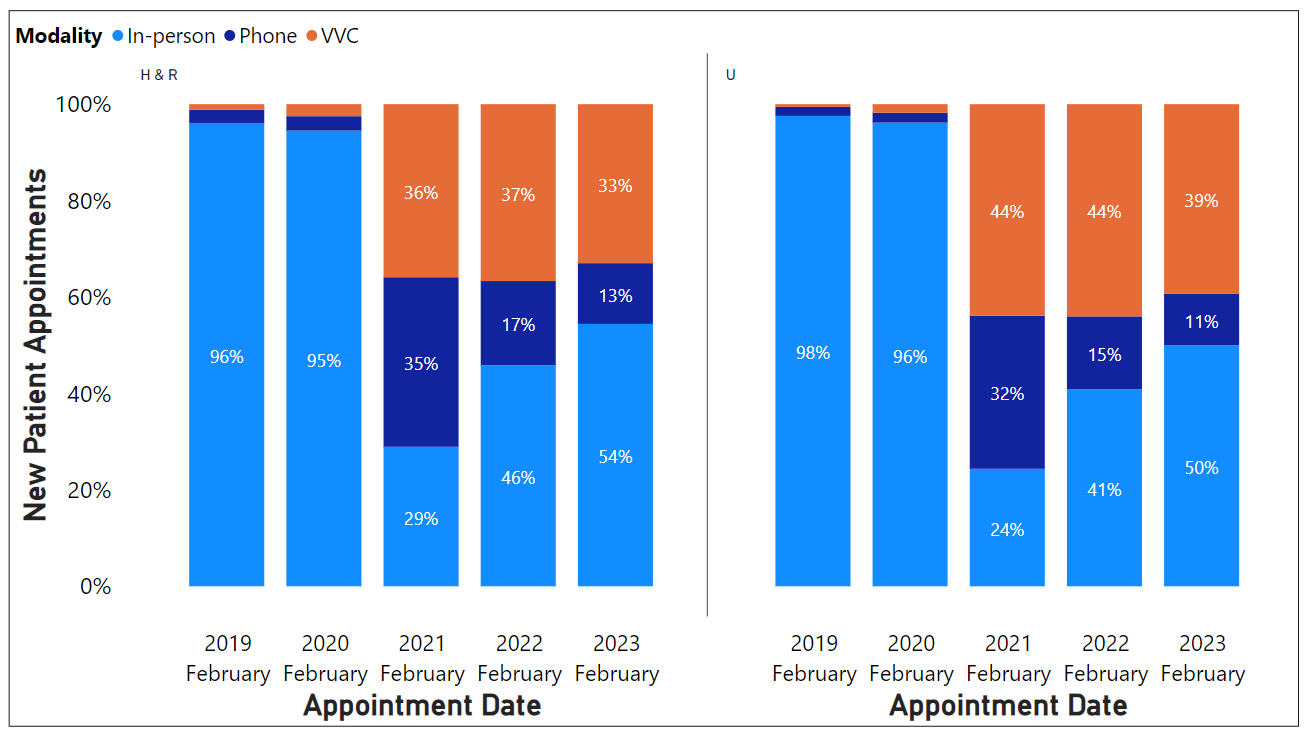


**Figure 12. Monthly new patient appointments for rural and highly rural patients (left), and urban patients (right).**


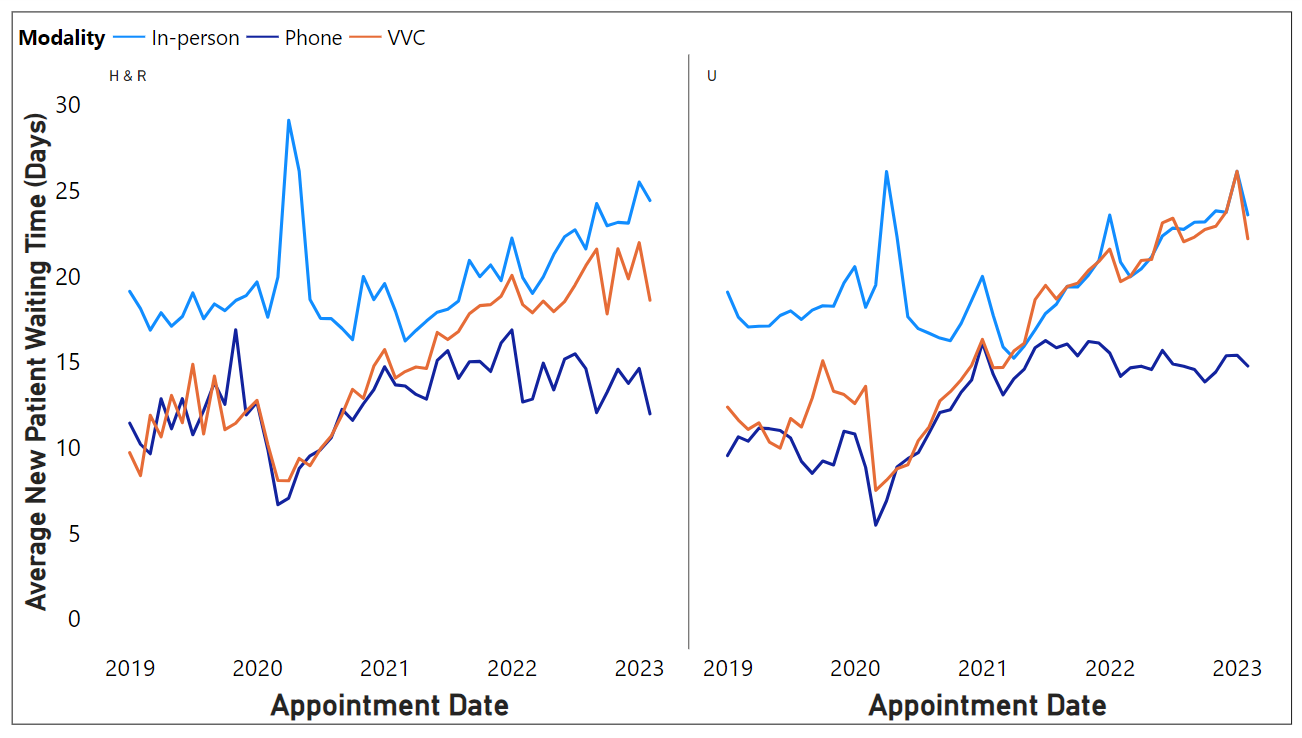


**Figure 13. Average monthly new patient waiting times for rural and highly rural patients (left), and urban patients (right).**

**
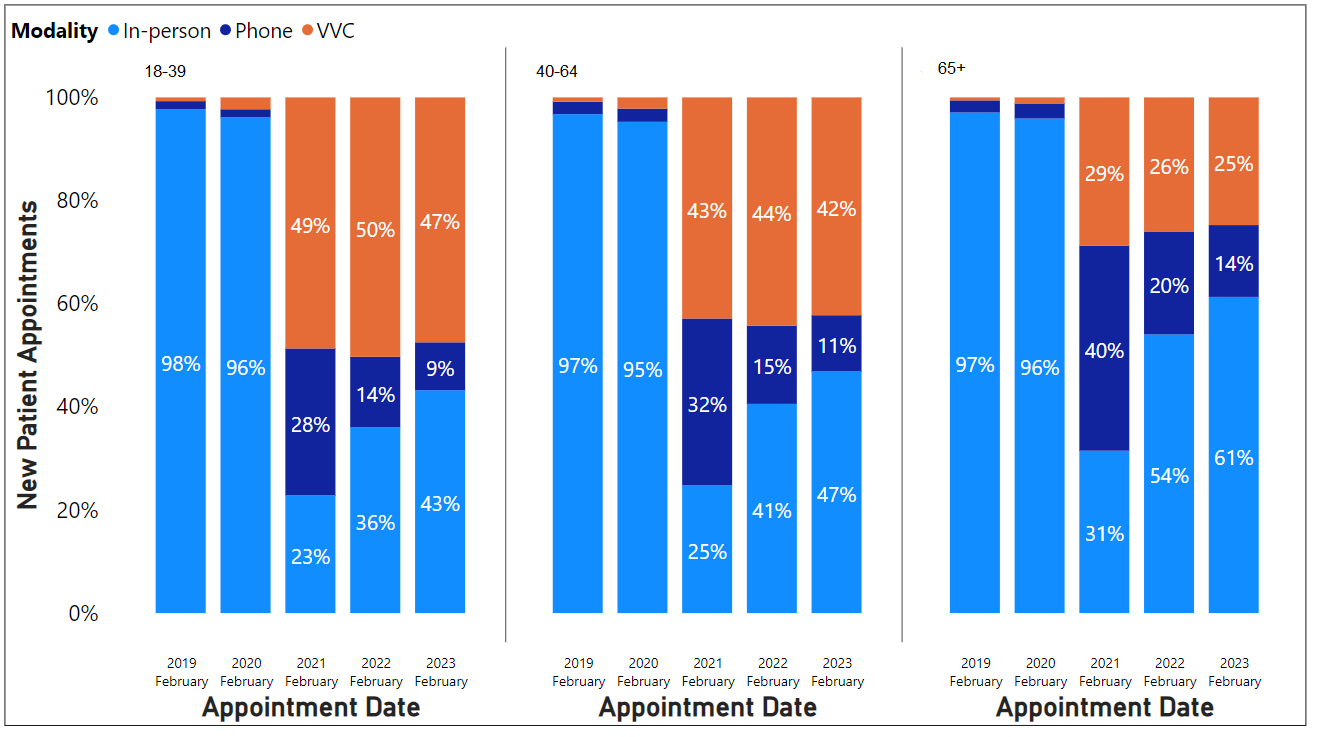
**

**Figure 14. Monthly new patient appointments for patients aged 18-39 (left), 40-64 (middle), and 65+ (right).**

***
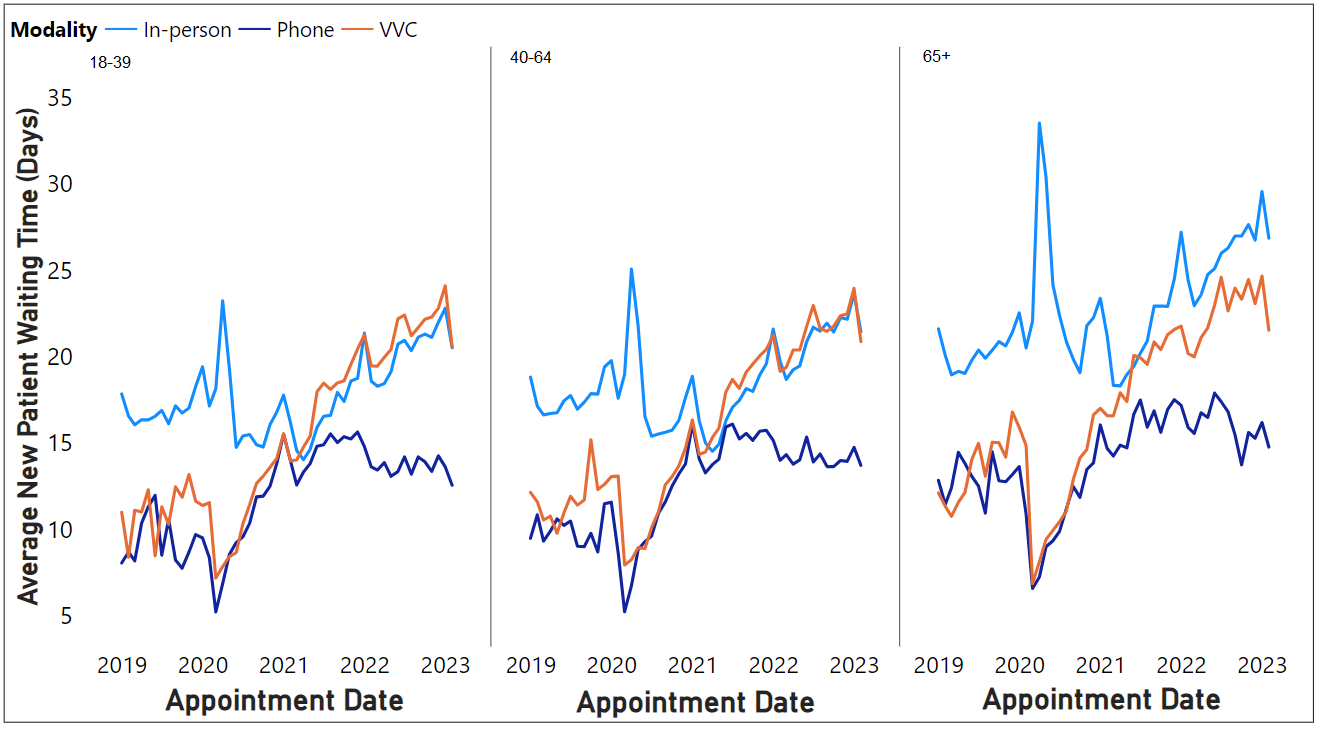
***

**Figure 15. Average monthly new patient waiting times for patients aged 18-39 (left), 40-64 (middle), and 65+ (right).**

**
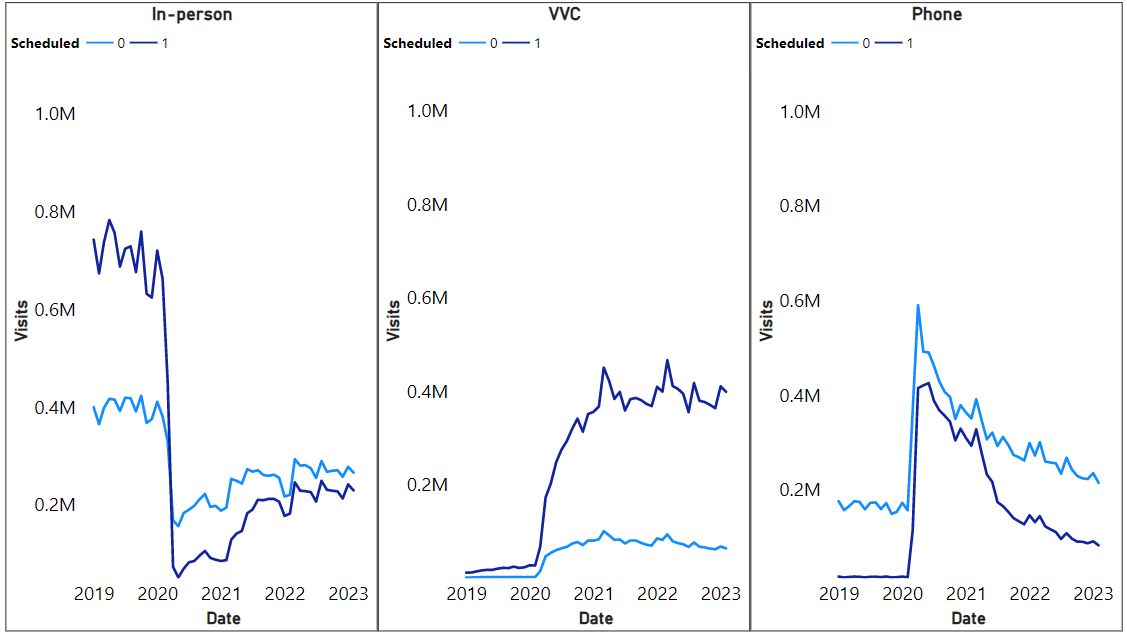
**

**Figure 16. Scheduled and unscheduled visit volumes across modalities.**

**Supplementary Table 1: Stop Codes used to categorize care type for encounters***

| **CareType** | **Stop Codes with Description** |
| --- | --- |
| Mental Health | 156 Hbpc - Psychologist, 157 Hbpc - Psychiatrist, 292 Observation Psychiatry, 502 Mental Health Clinic - Individual, 504 Grant & Per Diem - Group, 507 Hud/Vash - Group, 508 Hchv/Hcmi - Group, 509 Psychiatry, 510 Psychology, 511 Grant & Per Diem - Individual, 513 Substance Use Disorder - Individual, 516 Ptsd - Group, 522 Department Of Housing And Urban Development (Hud)-Va Supported Housing (Vash) Individual, 523 Opioid Treatment Program, 524 Active Duty Sexual Trauma, 525 Women's Stress Disorder Treatment Teams, 527 Telephone Mental Health, 528 Telephone Homeless Chronically Mentally Ill (Hcmi), 529 Hchv/Hcmi - Individual, 530 Telephone/Hud-Vash, 533 Mental Health Intervention Biomedical Care - Individual, 534 Mental Health Integrated Care - Individual, 535 Mental Health Vocational Assistance - Individual, 536 Telephone Mental Health Vocational Assistance, 538 Psychological Testing, 539 Mental Health Integrated Care - Group, 542 Telephone/Post-Traumatic Stress Disorder (Ptsd), 545 Telephone/Substance Use Disorder, 546 Telephone Intensive Community Mental Health Recovery Services (Icmhr), 547 Intensive Substance Use Disorder - Group, 550 Mental Health Clinic - Group, 552 Intensive Community Mental Health Recovery Services (Icmhr)- Individual, 555 Homeless Veteran Community Employment Services - Individual, 556 Homeless Veteran Community Employment Services - Group, 560 Substance Use Disorder - Group, 561 Pct-Post Traumatic Stress - Group, 562 Ptsd - Individual, 564 Mental Health Team Case Management, 565 Mental Health Intervention Biomedical Care - Group, 566 Mental Health Risk-Factor, Reduction Educational Group, 567 Intensive Community Mental Health Recovery Services (Icmhr)- Group, 568 Mental Health Compensated Work Therapy/ Supported Employment (Cwt/Se) , Face-To-Face, 573 Mental Health Incentive Therapy Face-To-Face, 574 Mental Health Compensated Work Therapy/Transitional Work Experience (Cwt/Twe) Face-To-Face, 575 Mental Health Vocational Assistance - Group, 576 Psychogeriatric Clinic - Individual, 577 Psychogeriatric Clinic - Group, 579 Telephone/Psychogeriatrics, 582 Psychosocial Rehabilitation Recovery Center (Prrc) - Individual, 583 Psychosocial Rehabilitation Recovery Center (Prrc)- Group, 584 Telephone Psychosocial Rehabilitation Recovery Center (Prrc), 586 Residential Rehabilitation Treatment Program (Rrtp) - Individual, 587 Residential Rehabilitation Treatment Program (Rrtp) - Group, 591 Incarcerated Veterans Re-Entry, 592 Veterans Justice Outreach, 593 Residential Rehabilitation Treatment Program (Rrtp) Outreach Services, 596 Residential Rehabilitation Treatment Program (Rrtp) Admission Screening Services, 597 Telephone/Residential Rehabilitation Treatment Program (Rrtp), 598 Residential Rehabilitation Treatment Program (Rrtp) Outpatient Individual, 599 Residential Rehabilitation Treatment Program (Rrtp) Outpatient Group |

**Supplementary Table 2: Stop Codes used to define care delivery method for encounters***

| Care Delivery Method | **Stop Codes with Description** |
| --- | --- |
| Video | 179: Real Time Clinical Video Telehealth To Home - Provider Site  648: Real Time Clinical Video Telehealth With Non-VAMC Location - Provider Site  679: National Center Real Time Clinical Video Telehealth To Home- Provider Site |
| Phone | 103 Telephone Triage, 147 Telephone/Ancillary,  178 Telephone Hbpc,  182 Telephone Case Management,  199 Telephone Polytrauma/Traumatic Brain Injury (Tbi),  216 Telephone/Rehabilitation (Rehab) And Support,  224 Telephone Spinal Cord Injury (Sci), 229 Telephone/Blind Rehab Program,  324 Telephone/Medicine,  325 Telephone/Neurology,  326 Telephone/Geriatrics,  338 Telephone Primary Care,  441 Telephone Anesthesia  527 Telephone Mental Health  528 Telephone Homeless Chronically Mentally Ill (Hcmi)  530 Telephone/Hud-VASH  536 Telephone Mental Health Vocational Assistance  542 Telephone/Post-Traumatic Stress Disorder (Ptsd),  545 Telephone/Substance Use Disorder  546 Telephone Intensive Community Mental Health Recovery Services (Icmhr)  579 Telephone/Psychogeriatrics,  584 Telephone Psychosocial Rehabilitation Recovery Center (Prrc)  597 Telephone/Residential Rehabilitation Treatment Program (Rrtp)  611 Telephone/Dialysis, 686 Telephone Contact By Home Telehealth (Ht) Staff  801 In-VISN, Other VAMC 2nd To 103  802 Out Of Visn, Va 2ndary To 103 |

*Figures from Ferguson, JM, Jacobs, J, Yefimova, M, Greene, L, Heyworth, L, & Zulman, DM. Virtual care expansion in the Veterans Health Administration during the COVID-19 pandemic: clinical services and patient characteristics associated with utilization. *Journal of the American Medical Informatics Association: JAMIA*. 2021; *28*(3), 453–462. https://doi.org/10.1093/jamia/ocaa284
